# Hypothalamic POMC neuron-specific knockout of MC4R affects insulin sensitivity by regulating Kir2.1

**DOI:** 10.1186/s10020-024-00804-z

**Published:** 2024-03-06

**Authors:** Hengru Guo, Ying Xin, Saifei Wang, Xiaoning Zhang, Yanqi Ren, Bo Qiao, Hongjiang Li, Jing Wu, Xiao Hao, Lijun Xu, Yushan Yan, Haohao Zhang

**Affiliations:** 1 Department of Endocrinology, The First Affiliated Hospital of Zhengzhou University, 450052 Zhengzhou, Henan China; 2 Department of Endocrinology, Third People’s Hospital, Zhengzhou, China; 3 Department of Neurosurgery, The First Affiliated Hospital of Zhengzhou University, Zhengzhou, China; 4 Department of Pediatrics, First Affiliated Hospital of Zhengzhou University, Zhengzhou, China

**Keywords:** MC4R, Arcuate nucleus, Kir2.1, Insulin resistance, Energy expenditure

## Abstract

**Background:**

Imbalance in energy regulation is a major cause of insulin resistance and diabetes. Melanocortin-4 receptor (MC4R) signaling at specific sites in the central nervous system has synergistic but non-overlapping functions. However, the mechanism by which MC4R in the arcuate nucleus (ARC) region regulates energy balance and insulin resistance remains unclear.

**Methods:**

The MC4R^flox/flox^ mice with proopiomelanocortin (POMC) -Cre mice were crossed to generate the POMC-MC4R^flox/+^ mice. Then POMC-MC4R^flox/+^ mice were further mated with MC4R^flox/flox^ mice to generate the POMC-MC4R^flox/flox^ mice in which MC4R is selectively deleted in POMC neurons. Bilateral injections of 200 nl of AAV-sh-Kir2.1 (AAV-sh-NC was used as control) were made into the ARC of the hypothalamus. Oxygen consumption, carbon dioxide production, respiratory exchange ratio and energy expenditure were measured by using the CLAMS; Total, visceral and subcutaneous fat was analyzed using micro-CT. Co-immunoprecipitation assays (Co-IP) were used to analyze the interaction between MC4R and Kir2.1 in GT1-7 cells.

**Results:**

POMC neuron-specific ablation of MC4R in the ARC region promoted food intake, impaired energy expenditure, leading to increased weight gain and impaired systemic glucose homeostasis. Additionally, MC4R ablation reduced the activation of POMC neuron, and is not tissue-specific for peripheral regulation, suggesting the importance of its central regulation. Mechanistically, sequencing analysis and Co-IP assay demonstrated a direct interaction of MC4R with Kir2.1. Knockdown of Kir2.1 in POMC neuron-specific ablation of MC4R restored the effect of MC4R ablation on energy expenditure and systemic glucose homeostasis, indicating by reduced body weight and ameliorated insulin resistance.

**Conclusion:**

Hypothalamic POMC neuron-specific knockout of MC4R affects energy balance and insulin sensitivity by regulating Kir2.1. Kir2.1 represents a new target and pathway that could be targeted in obesity.

**Supplementary Information:**

The online version contains supplementary material available at 10.1186/s10020-024-00804-z.

## Background

Imbalance in energy regulation is the main cause of insulin resistance (IR) and diabetes (Magkos et al. 2020). Melanocortin-4 receptor (MC4R) has drawn much attention not only because it is the most common target of mutations causing monogenic obesity but also because it remains an important drug target for other forms of obesity as well (Clément et al. 2018; Wei et al. 2023). The features of the MC4R structure that are critical for ligand binding have evolved to allow regulation by two unrelated endogenous ligands: the linear tridecapeptide agonistα-melanocyte-stimulating hormone (α-MSH), which activates MC4R and leads to reduced appetite, and the 50-amino acid cystine-knot antagonist or biased agonist agouti-related protein (AgRP), which leads to increased food intake. It is reported that subcutaneous injection of MC4R agonists can increase peripheral energy expenditure and insulin sensitivity (Kühnen et al. 2016). MC4R deficiency in mice is associated with insulin resistance, renal sympathetic nerve activity inhibition and glucosuria elevation (de Souza Cordeiro et al. 2021). Furthermore, hyperinsulinemic euglycemic clamp experiments demonstrated that targeted depletion of MC4R in autonomic preganglionic fibers resulted in decreased glucose infusion rates and decreased glucose uptake in skeletal muscle and adipose tissue in the scapular region (Berglund et al. 2014). MC4R is widely expressed in the central nervous system, such as the paraventricular nucleus, dorsomedial nucleus, lateral hypothalamus and ARC (Kishi et al. 2003). Previous studies have identified distinct roles of MC4R signaling in specific neural circuits (e.g., paraventricular nucleus (PVN), dorsomedial, lateral region, lateral parabrachial nucleus) (Han et al. 2021; Liu et al. 2023; Morgan et al. 2015), suggesting that MC4R signaling at specific sites in the central nervous system is synergistic but non-overlapping functions. For instance, restoring MC4R expression in the PVN greatly reduced food intake (Singh et al. 2022). MC4R in the single-minded 1 (SIM1)-expressing PVN neurons mediated satiety and bodyweight by a PVN (MC4R)-lateral parabrachial nucleus pathway (Garfield et al. 2015; Singh et al. 2022). In ARC nucleus, MC4R agonists depolarize POMC neurons, modulating the excitability of arcuate POMC neurons by alteration of resting potassium conductances (Smith et al. 2007). Besides that, Kir7.1 was regulated by MC4R through a mechanism independent of Gαs and cAMP (Litt et al. 2018). However, the mechanism by which MC4R in the ARC region regulates energy balance and insulin resistance remains unclear. Bagnol et al. (Bagnol et al. 1999) found that there was no distribution of AgRP neuron terminals in the presympathetic motor area of the brainstem by immunohistochemical experiments. Therefore, whether POMC neurons in the ARC region regulate energy metabolism and insulin resistance through sympathetic nerves remains to be explored.

Inwardly rectifying potassium channels are an important class of potassium channels, which are characterized by opening when the membrane is hyperpolarized and producing inward potassium currents, which are mainly involved in maintaining the resting membrane potential and hyperpolarization of cells, and directly affect the action potential, time period and morphology, and thus play an important role in the regulation of neuronal activity (Ferreira et al. 2023; Martelli 2018). It is reported that Ba^2+^, a non-selective inward rectifier potassium (KIR) channel blocker, induced a small depolarization of POMC neurons (Smith et al. 2007). Kir2.1, a strong inward rectifier potassium channel encoded by the KCNJ2 gene, is a key regulator of the resting membrane potential of the cardiomyocyte and plays an important role in controlling ventricular excitation and action potential duration in the human heart (Park et al. 2020). Kir2.1 is widely distributed in the whole brain and can regulate cell excitability, maintain resting membrane potential and neurotransmitter secretion (Negri et al. 2021). It has been reported that Kir2.1 is associated with the occurrence and development of diabetes (Law et al. 2020). Blocking of Kir2.1 channels augments the rate of insulin secretion in human β-cells, while hyperactive Kir2.1 channels may lead to reduced insulin secretion (Morgan et al. 2015). In pyramidal neurons, overexpression of Kir2.1 inhibits neuronal activity (Xue et al. 2014). Regulation of G protein-coupled inwardly rectifying potassium (GIRK) channels by G protein-coupled receptors (GPCRs) via the G protein βγ subunits has been well characterized (Carrington et al. 2018). However, whether MC4R regulates Kir2.1 is incompletely understood.

Increased excitability and axonal projections of POMC neurons located in the arcuate nucleus can reduce appetite, accelerate metabolism and energy expenditure (Vohra et al. 2022). This project aims to reveal the role of POMC neurons in the ARC region in regulating energy balance and insulin resistance. Taken together, we speculate that MC4R-specific knockout in the arcuate nucleus upregulates Kir2.1 expression and inhibit POMC neuron activity, thereby increasing intake and insulin resistance in mice.

## Materials and methods

### Mice and diets

MC4R^flox/+^ mice (NM-CKO-200195) were purchased from the Shanghai Model Organisms (Shanghai, China). POMC-Cre mice (stock number #005965) were purchased from the Jackson laboratory (Bar Harbor, ME, USA). To obtain POMC neuron-specific MC4R knockdown and littermate wildtype (WT) mice, we crossed the MC4R^flox/+^ mice with POMC-Cre mice to generate POMC-MC4R^flox/+^ mice, as well as self-crossed the MC4R^flox/+^ mice to generate MC4R^flox/flox^ mice. The POMC-MC4R^flox/+^ mice were further mated with MC4R^flox/flox^ mice to generate the POMC-MC4R^flox/flox^ mice in which MC4R is selectively deleted in POMC neurons (the littermate MC4R^flox/flox^: Cre^−^ mice were used as control). Tail biopsies and brain tissue were harvested and a PCR assay was performed to verify genotyping. Besides, immunofluorescent staining assay was used to detect the knockdown of MC4R in POMC neuron.

The animals were singly housed under a standard condition with a 12:12 h light-dark cycle at 25 ℃ with free access to water. Chow (9.4% kcal from fat, #SPF-F02-001) and high-fat (60% kcal from fat, #D12492) diets were purchased from the SPF biotechnology (Beijing, China). The chow-treated mice were fed regular chow throughout the experiment. The HFD-treated mice were subjected to regular chow for the first eight weeks, followed by a HFD for three months (Supplementary Fig. 1). All animal experiments were approved by the Ethics Committee of the First Affiliated Hospital of Zhengzhou University (2021-KY-0906-001).

### Adeno-associated viruses (AAV)-sh-Kir2.1 injections

POMC-MC4R^flox/flox^ mice were anesthetized with isoflurane and placed on a stereotaxic apparatus (David Kopf Instruments, Tujunga, CA). The skull was exposed via a small incision, and a small hole was drilled (0.45-mm drill bit) into the skull. A Hamilton 5-µl syringe with a 30-gauge blunt-end needle was inserted into the brain for the delivery of Kir2.1 shRNA adeno-associated viruses (AAVs, 1 × 10^12^ v.g/ml, Genechem, Shanghai, China). Bilateral injections of AAVs (200 nl of AAV-sh-Kir2.1 or AAV-sh-NC) were made into the ARC of the hypothalamus (Coordinates: 1.46 mm posterior to and 0.26 mm lateral to bregma, as well as 5.80 mm below the surface of skull). Mice were allowed to recover for 10 days.

### Primary hypothalamic neuron isolation and treatments

Primary hypothalamic neurons were isolated from fetal mice, embryonic day 18 using Neuron Isolation Kit, according to the manufacturer’s instructions (Miltenyi Biotec, Bergisch-Gladbach, Germany) and were cultured in neurobasal medium (Gibco). Briefly, after the pregnant mice were killed, the fetal mice were quickly removed from the mother, decapitated and the brain was isolated. The hypothalamus was dissected along the anterior border of the optic chiasm, posterior border of the mammillary body, upper border of the anterior commissure, and lateral border halfway from the lateral sulcus in the ventral side of brain. Subsequently, the neurons were isolated from hypothalamus using Neuron Isolation Kit, according to the manufacturer’s instruction. Primary neurons were infected with MC4R overexpression adenovirus or MC4R knockdown adenovirus, and cells were harvested 48 h after transfection.

### Metabolic parameter detection and fat level

Oxygen consumption, carbon dioxide production, respiratory exchange ratio (RER), energy expenditure, physical activity, meal frequency, meal size, meal duration and meal interval were measured by using the CLAMS (Columbus Instruments, Columbus, OH). Total, visceral and subcutaneous fat was analyzed using Micro-CT (Bruker SkyScan 1276, Bremen, Germany).

### Intraperitoneal glucose tolerance test (IPGTT)

Mice were fasted overnight and then intraperitoneally administered 20% glucose (w/v, 2 g/kg body weight). Blood was squeezed out from the tail vein and centrifuged at 4000 rpm for 15 min. Blood glucose level was measured by using a glucometer (Roche, Basel, Switzerland) at the indicated time points (0, 15, 30, 60, 90, 120 min).

### Intraperitoneal insulin tolerance test (IPITT)

For the detection of plasma insulin level, mice were fasted 4 h and then intraperitoneally administered insulin (0.6 U/kg body weight). Blood was collected from the tail vein and centrifuged at 4000 rpm for 15 min. Plasma insulin was detected using the Mercodia Ultrasensitive Insulin ELISA kit (ALPCO Diagnostic) at the indicated time points (0, 15, 30, 60, 90, 120 min).

### Immunofluorescence

Mice were anesthetized, and then fixed with 4% paraformaldehyde (PFA) via transcardial perfusion. Brain tissues were cryoprotected with sucrose solutions and sectioned on a cryostat (Leica, Wetzlar, Germany). Tissue sections were fixed with 4% formaldehyde for 15 min, and then permeabilized with 0.5% Triton X-100 for 20 min. After blocking with normal goat serum in 0.1% PBS-Tween for 30 min, tissue sections were incubated with primary antibodies overnight at 4 °C. The tissue sections were then incubated with Goat anti-Mouse IgG (H + L) Highly Cross-Adsorbed Secondary Antibody, Alexa Fluor™ Plus 488 (4 µg/ml, #A32723, Invitrogen, Carlsbad, CA, USA) and Goat anti-Rabbit IgG (H + L) Highly Cross-Adsorbed Secondary Antibody, Alexa Fluor™ 594 (4 µg/ml, #A-11037, Invitrogen) at 1:2000 dilution each at room temperature for 1 h. Slides were counterstained with DAPI for 5 min in the dark to show cell nuclei. Images were captured at 40 × magnification with a FluoView FV1200 confocal microscope (Olympus, Tokyo, Japan), processed and analyzed with the ImageJ software (Ver. 1.8, NIH, Bethesda, MD). Cells were manually counted in one side of the Arc nucleus in a representative image acquired for each mouse. The primary antibodies are as follows: rabbit anti-POMC antibody (1:1000, #23499S, Cell Signaling Technology Danvers, MA, USA), mouse anti-MC4R antibody (1:1000, #Sc-55567, Santa Cruz Biotechnology, CA, USA), Kir2.1 mouse monoclonal (5 µg/ml, #Ab85492, Abcam, Cambridge, UK), and c-FOS mouse monoclonal (1:800, #Ab208942, Abcam).

### Quantitative RT-PCR

Tissue RNA was extracted by using the TRIzol reagent (Thermo Fisher). Complement DNA was generated with MMLV reverse transcriptase (Promega, Madison, WI). PCR was performed on an ABI 7900HT thermocycler (Thermo Fisher) after the combination of cDNA, primers and master mix. We used the 2^−ΔΔCt^ method to analyze the relative expression of genes. The primers for MC4R are 5’- CCCGGACGGAGGATGCTAT-3’, 5’- TCGCCACGATCACTAGAATGT-3’. The primers for the housekeeping gene GAPDH are forward 5’- ACTCTTCCACCTTCGATGC − 3’ and reverse 5’- CCGTATTCATTGTCATACCAGG − 3’.

### Western blot analysis

BAT, liver, soleus and hypothalamic tissues were collected from MC4R WT and KO mice fed a HFD for 13 weeks at the age of 8 weeks. After the tissues were removed, they were cut up into 1 cm^3^, digested in trypsin for 30 min, and then added to the medium containing serum to terminate digestion. Protein extracts were then prepared using RIPA buffer, and the concentrations were determined by using a BCA protein assay kit (Pierce, Rockford, IL, USA). Fifty micrograms of lysates were separated by SDS polyacrylamide 10% gradient gel (Sodium dodecyl sulphate/polyacrylamide gel), transferred to a polyvinylidene difluoride membrane (PVDF membrane, Millipore, Boston, MA, USA). The membranes were blocked with 5% nonfat dry milk in Tris-buffered saline, pH 7.4, containing 0.05% Tween 20, and were incubated with primary antibodies and horseradish peroxidase-conjugated IgG antibodies (1:5000, Santa Cruz) according to the manufacturer’s instructions. The protein of interest was visualized using enhanced chemiluminescence (ECL) system (Solarbio,Beijing, China). The primary antibodies are as follows: anti-MC4R (1:2000, Sc-55567, Santa Cruz Biotechnology, Santa Cruz, CA), anti-Kir2 (1 µg/ml, Ab85492, Abcam), IR (1:1000, 07-724, Millipore), p-IR (1:1000, 44800G, Invitrogen), AKT (1:1000, 9272 S, Cell Signaling Technology, Danvers, MA, USA), p-AKT (1:2000, 4060T, Cell Signaling Technology), AgRP (1:1000, ab113481, Abcam), NPY(1:1000, D7Y5A, Cell Signaling Technology), leptin (1:1000, PA1-051,Thermo Fisher), MC1R (1:2000, Ab180776, Abcam), MC3R (1:2000, STJ27686, St John’s Labs), MC5R (1:200, AMR-025, Alomone Labs) and GLP-1(1:2000, Ab36598, Abcam).

### Transcriptome analysis

MC4R overexpressed adenovirus-transfected GT1-7 cells (empty vector adenovirus-transfected GT1-7 cells were used as control) were used for transcriptome analysis. The GT1-7 cells were sent to Wuhan BGI Technology Co., Ltd. (Hubei, China) for high-throughput sequencing using the BGISEQ-500 platform. RNA differential expression analysis was performed using DESeq2 software between two different groups. Transcripts with a false discovery rate (FDR) below 0.05, and absolute fold change ≥ 2 were considered differential expression genes.

### Co-immunoprecipitation assays

GT1-7 cells (1 × 10^7^) were donated by the Clinical Center of Endocrinology and Metabolism of Shanghai Ruijin Hospital were washed with ice-cold PBS, and lysed in 20 mM Tris-HCl, pH 8.0, 150 mM NaCl, 1 mM EDTA, 1% Nonidet P-40 supplemented with protease inhibitor PMSF on ice for 30 min. After centrifuged at 12,000 rpm for 15 min at 4℃, the supernatant was collected and 20 µl of supernatant was used as Input. The remaining cell lysates supernatant was immunoprecipitated with antibody against Kir2.1 (20 µg/ml) at 4℃ overnight. The immunocomplex was captured with protein A-agarose beads (Invitrogen, normal mouse IgG was used as negative control), and incubated for 4 h at 4℃. Then, the complex were washed with washing buffer for three times, and analyzed by Western blotting.

### Statistical analysis

All statistical analyses involved use of SPSS20.0 (SPSS Inc, Chicago, IL). Data are reported as the mean ± standard deviation (SD). For 2-group comparisons of parametric data, a Student *t* test was performed, whereas nonparametric data were analyzed with the Mann-Whitney test. Statistical significance between multiple groups was determined by one‐way ANOVA followed by Dunnett's post hoc test or two-way ANOVA. *P* < 0.05 was considered statistically significant.

## Results

### POMC neuron-specific deletion of MC4R leads to obesity and decreases energy expenditure

**Fig. 1:**
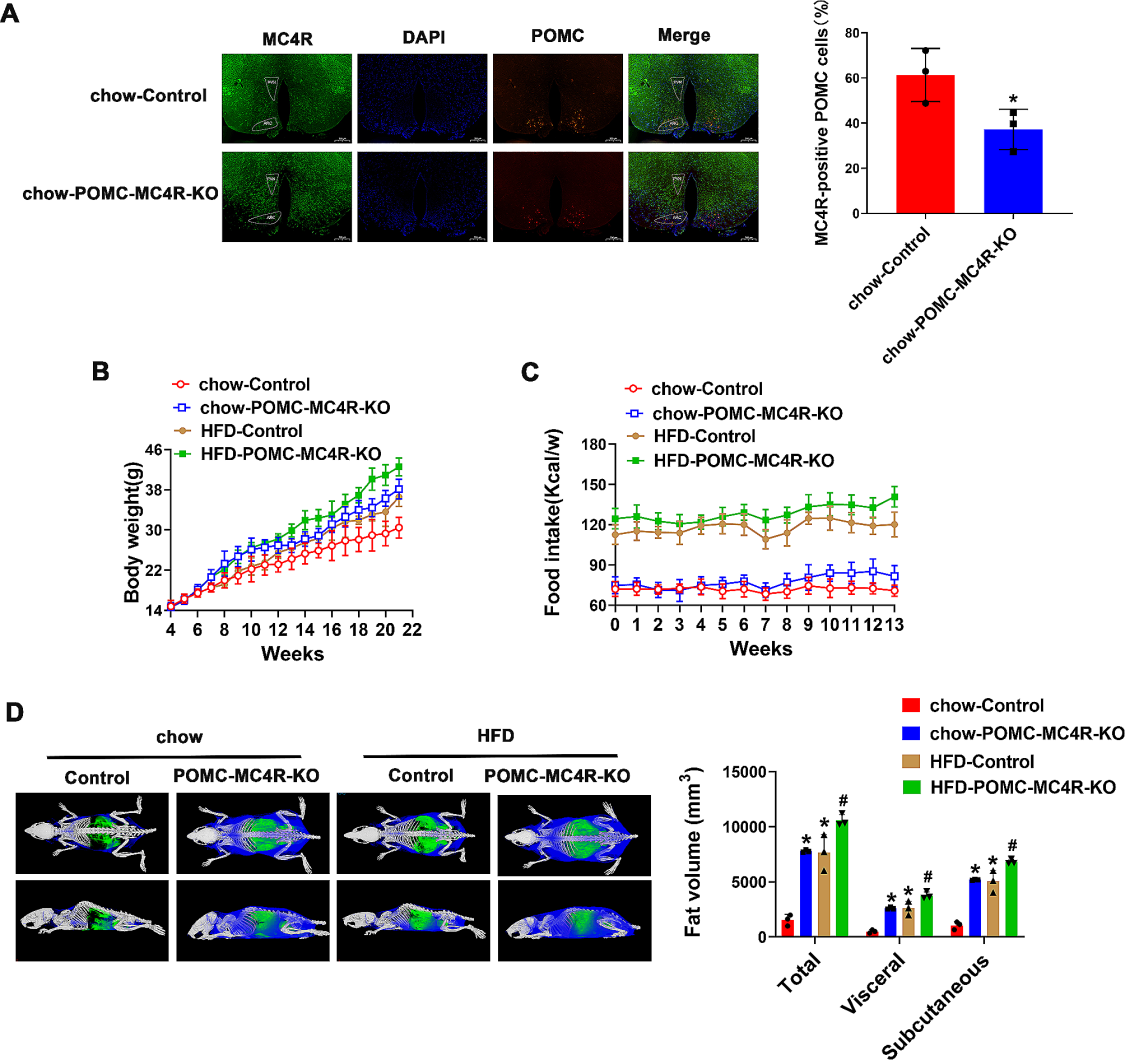
Ablation of MC4R in the hypothalamus leads to obesity. (**A**) Immunoflurescent staining for POMC and MC4R of the hypothalamus of POMC-MC4R^flox/flox^ mice. Scale bar = 500 μm above the picture, Scale bar = 200 μm below the picture. (**B**) Body-weight in MC4R WT and KO mice fed a chow or HFD from 8 wk of age. (**C**) Food intake assessed during the first 13 wk of diet treatment. (**D**) Fat mass at 21 wk old. Data are presented as means ± SD. *P < 0.05, **P < 0.01, two-tailed Student’s *t* test was used for comparison for (**A**) MC4R-positive POMC cells; two-way ANOVA for (**D**) fat volume. Repeated measures ANOVA for multiple comparisons for (**B**) body weight and (**C**) food intake

**Fig. 2:**
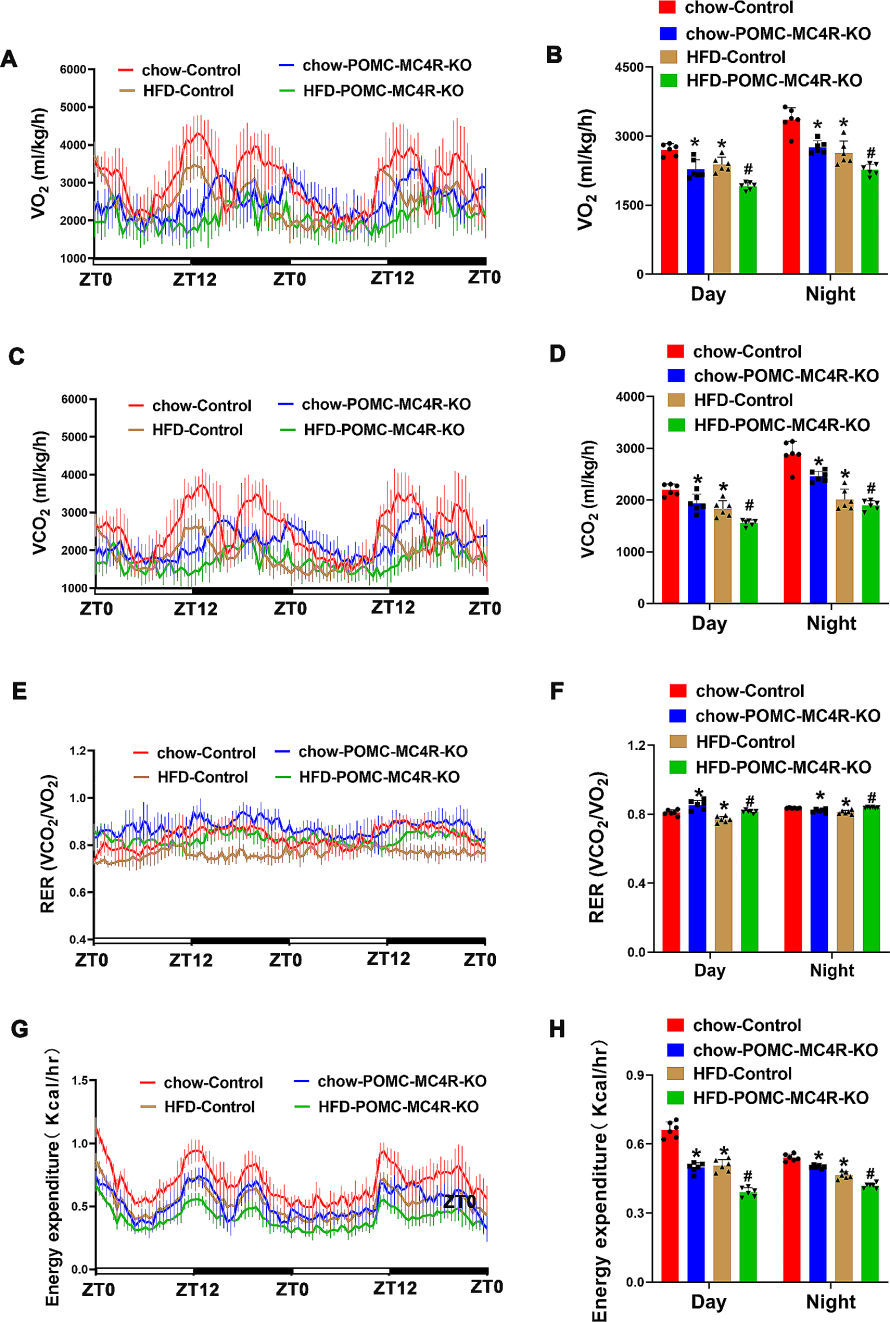
Effect of MC4R ablation on energy balance. (**A** and **B**) O_2_ consumption (VO_2_), (**C** and **D**) CO_2_ production, and (**E** and **F**) RER. (**G** and **H**) Energy expenditure was evaluated of the mice. Data are presented as means ± SD. *P < 0.05, **P < 0.01; two-way ANOVA for (**B**) VO_2_, (**D**) CO_2_ production, (**F**) RER, and (H) Energy expenditure

It is established that MC4R not only expressed in the PVN, but also highly expressed in ARC and ventromedial hypothalamic nucleus (Wang et al. 2020). We generated POMC neuron-specific MC4R knockdown (POMC-MC4R^flox/flox^) and littermate control (control, MC4R^flox/flox^:Cre^−^) mice. The genotyping of mice was validated by PCR analysis (Supplementary Fig. 2A). Immunofluorescent staining assay demonstrated that MC4R protein was efficiently eliminated from the POMC neurons (Fig. 1A). As expected, the percentage of MC4R-expressing POMC neurons in the ARC was significantly reduced in the POMC-MC4R^flox/flox^ mice (61.27 ± 11.73% for the control mice, and 37.18 ± 8.89% for the POMC-MC4R^flox/flox^ mice). However, in both control and POMC-MC4R^flox/flox^ mice, MC4R is highly expressed in PVN region (Supplementary Fig. 2B-C). At the age of 8 wk, chow-Control and chow-POMC-MC4R-KO mice did not differ in body weight. These mice were then subjected to a HFD, or maintained on the regular chow. Under this condition, POMC-MC4R^flox/flox^ mice exhibited a remarkably increased body weight from the age of 13-weeks old compared with control mice, accompanied by an increased food intake (Fig. 1D), indicating that MC4R is involved in developing of obesity. This effect was mainly ascribed to the increment in fat mass (Fig. 1D). Decreased oxygen consumption (Fig. 2A-B) and CO_2_ consumption (Fig. 2C-D), while elevated RER (VCO_2_/ VO_2_; Fig. 2E-F) was found in POMC-MC4R^flox/flox^ mice. Moreover, POMC-MC4R^flox/flox^ mice exhibited lower energy expenditure (Fig. 2G-H) and physical activity (Supplementary Fig. 3A-B). HFD-Control mice showed an increase in meal frequency at 8–9 weeks of age, whereas meal size, meal duration, and meal interval decreased. For 20–21 week old mice, meal frequency and meal duration were decreased, whereas meal size and meal interval were increased in HFD-Control mice (Supplementary Fig. 3C-F). MC4R deletion had no significant effect on meal frequency, but it did increase meal size and meal duration (Supplementary Fig. 3C-F). Taken together, these data support a significant role for POMC-MC4R in the development of HFD.

### Deletion of MC4R in the hypothalamus reduces insulin sensitivity

**Fig. 3:**
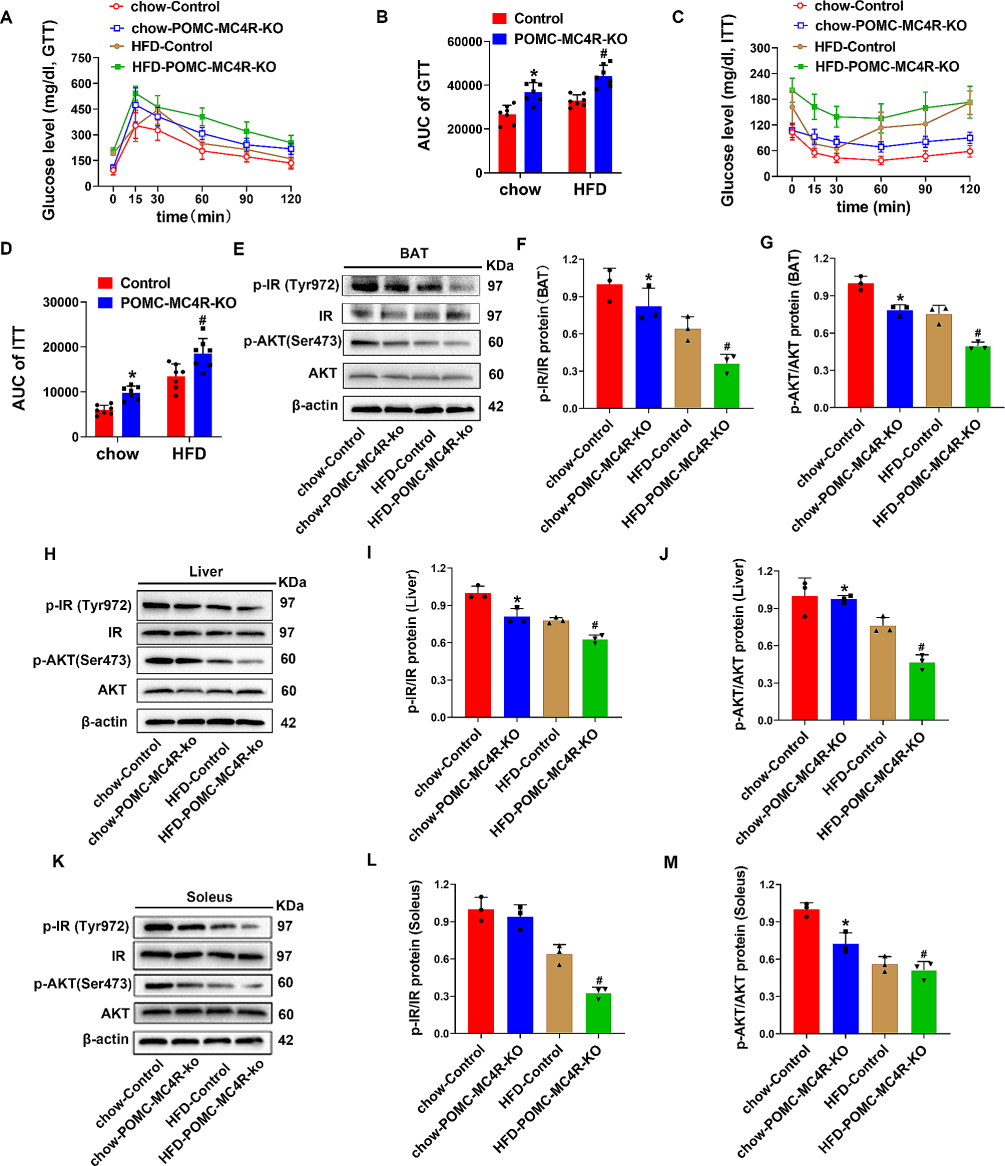
Ablation of MC4R in the hypothalamus decreased hepatic insulin sensitivity. (**A** and **B**) GTT (**A**) and the AUC of GTT (**B**) of 21-wk-old mice. (**C** and **D**) ITT (**C**) and the AUC of ITT (**D**) of 21-wk-old mice. (**E**, **F** and **G**) Relative protein expressions of IR and Akt were analyzed by western blot in BAT. (**H**, **I** and **J**) Relative protein expressions of IR and Akt were analyzed by western blot in liver. (**K**, **L** and **M**) Relative protein expressions of IR and Akt were analyzed by western blot in soleus. Two-way ANOVA for (**B**) AUC of GTT, (**D**) AUC of ITT, (**F**, **I**, **L**) p-IR/IR protein, and (**G**, **J**, **M**) p-AKT/AKT. Repeated measures ANOVA for multiple comparisons for (**A**) glucose level (GTT) and (**C**) glucose level (ITT). *P < 0.05 vs. chow-Control; ^#^*P* < 0.05 vs. HFD-Control.

We then examined the effect of hypothalamic MC4R deletion on insulin resistance. GTT data and AUC of the GTT suggested that glucose tolerance was greatly reduced in MC4R deletion mice compared with the control group (Fig. 3A-B). Additionally, MC4R deletion mice induced attenuated glucose clearance, as indicated by increased ITT level and AUC of the ITT (Fig. 3C-D). The neural connection between the hypothalamus and the brown adipose tissue (BAT), liver, and soleus plays an important role in the CNS control of systematic glucose homeostasis. Therefore, the phosphorylation levels of two key components in the insulin signaling pathways, insulin receptor (IR) and protein kinase B (AKT), in the liver, BAT, and soleus after infusion of insulin (2 units/kg) into the hepatic portal vein was analyzed. Importantly, deletion of MC4R also significantly reduced insulin-stimulated phosphorylation of IR and AKT in the BAT compared with the control group (Fig. 3E-G). The phosphorylation of IR and AKT were also decreased in liver (Fig. 3H-J) and soleus (Fig. 3K-M) in MC4R deletion mice compared to that in the wild-type mice. Furthermore, compared with the chow-Control group, there was a significant increase in AgRP, NPY, Leptin, MC3R, as well as a decrease in GLP-1 expression in chow-POMC-MC4R-KO mice, suggesting a potential compensatory mechanism in response to MC4R loss (Supplementary Fig. 4A-H).

### Knockdown of MC4R inhibits activation of POMC neurons

**Fig. 4:**
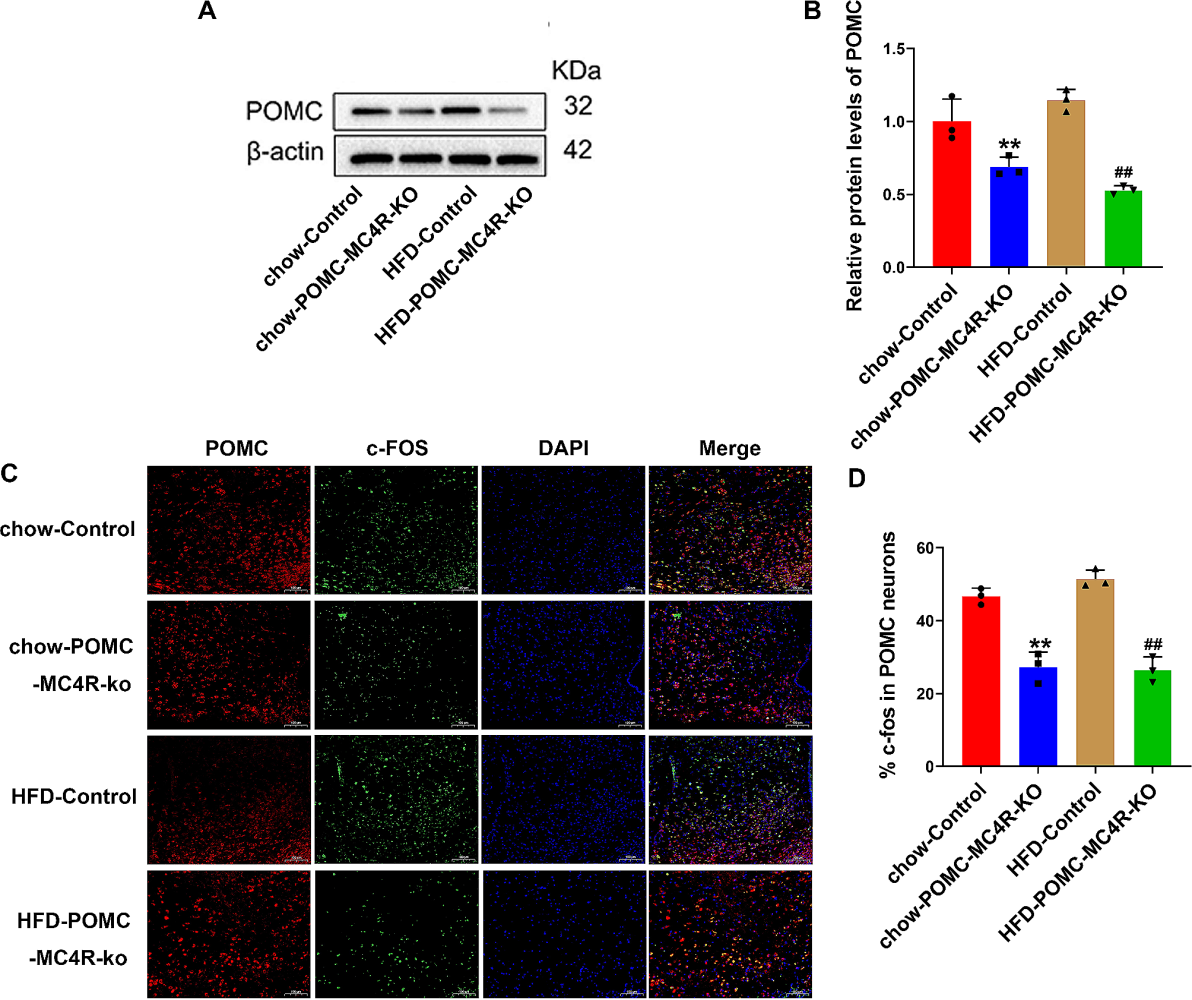
Ablation of MC4R drives activation of POMC neurons. (**A** and **B**) POMC western blot and densitometric quantification in hypothalamus. (**C** and **D**) Immunofluorescence for POMC neurons (red), c-Fos (green) and merge in ARC sections and integrated density quantification in POMC neurons and colocalization in 21-weeks old control and MC4R mice treated with or without HFD. Scale bar = 100 μm. Two-way ANOVA. *P < 0.05 vs. chow-Control; ^#^*P* < 0.05 vs. HFD-Control.

To determine the effect of MC4R knockdown on POMC neuronal activation, we performed and quantified c-Fos immunostaining in the POMC of MC4R knockdown and control mice. The protein expression of POMC was significantly decreased in POMC-MC4R^flox/flox^ mice (Fig. 4A-B). Meanwhile, a significant decrease of c-Fos immunoreactivity was found in HFD-POMC-MC4R-KO mice (26.55 ± 3.50 c-Fos in POMC neurons) compared to HFD-Control mice (51.40 ± 2.48 c-Fos in POMC neurons; Fig. 4C-D). These results suggested that knockdown of MC4R in ARC neurons resulted in inactivation of POMC neurons.

### MC4R negatively regulates the expression of Kir2.1

**Fig. 5:**
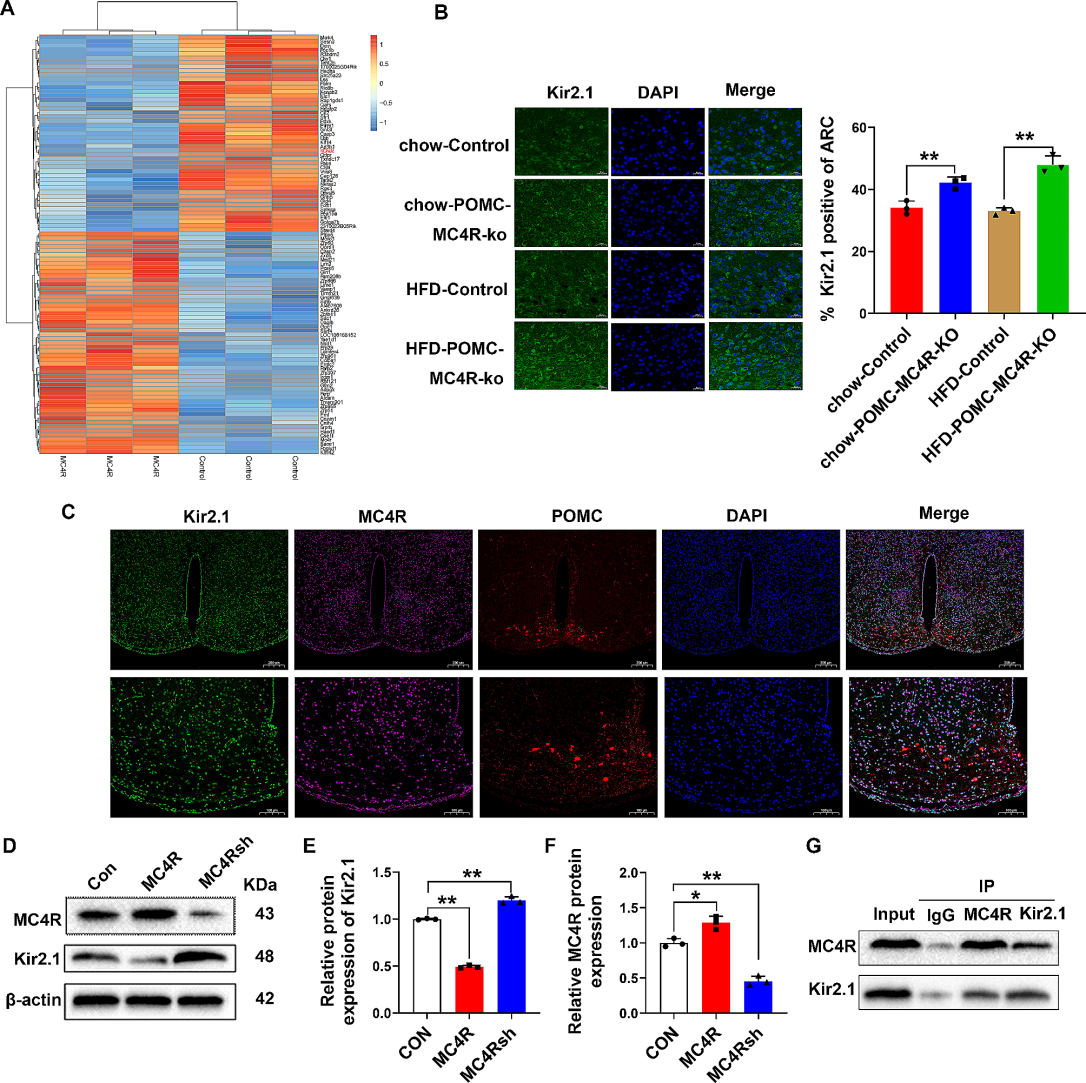
MC4R negatively regulates the expression of Kir2.1. (**A**) Transcriptome analysis found that the expression of potassium inwardly-rectifying channel subfamily J member 2, KCNJ2, or Kir2.1 was down-regulated. (**B**) Immunofluorescence for Kir2.1 (green) and merge. Scale bar = 20 μm. (**C**) Immunofluorescence for Kir2.1 (green), MC4R (purple) and POMC (red) in HFD mice. Scale bar = 200 μm at the top, Scale bar = 100 μm at the bottom. (**D**-**F**) Primary hypothalamic neurons were transfected with MC4R shRNA plasmid, and the transfection efficiency and the protein expression of Kir2.1 were analyzed by western blot. (G) Co-IP assay detected the interaction of MC4R and Kir2.1. *P < 0.05, **P < 0.01. One-way ANOVA with Dunnett's post hoc test for (**D**) relative MC4R protein expression and (**E**) relative protein expression of Kir2.1. Two-way ANOVA for (B) % Kir2.1 positive of ARC.

Overexpression of MC4R in mouse hypothalamic GT1-7 cells, and transcriptome analysis found that the expression of potassium inwardly-rectifying channel subfamily J member 2, KCNJ2, or Kir2.1 was down-regulated (Fig. 5A). Immunostaining assay revealed that Kir2.1 positive cell was increased in chow-POMC-MC4R-KO mice when compared to the chow-Control mice, as well as increased in HFD-POMC-MC4R-KO mice when compared to the HFD-Control mice (Fig. 5B). Besides that, Kir2.1 and MC4R are colocalized in POMC neurons in the ARC (Fig. 5C). Subsequently, we utilized primary hypothalamic neurons to address the relationship among MC4R and Kir2.1 genes. Cells were transfected with MC4R overexpression or MC4R knockdown plasmids (Supplementary Fig. 5). Western blots confirmed that they successfully increased or decreased the protein levels of MC4R (Fig. 5D-E). The results showed that overexpression of MC4R significantly decreased, whereas knockdown of MC4R increased the level of Kir2.1 (Fig. 5D and F). We also used co-immunoprecipitation assays to determine if MC4R associates directly with the Kir2.1 in an in vitro neuronal cell line GT1-7 cells. Co-immunoprecipitation assay using anti-Kir2.1antibody revealed that Kir2.1 co-precipitated with MC4R (Fig. 5G). Collectively, these data suggest that MC4R has a negative role in controlling the transcriptional activities of Kir2.1 gene.

### Knockdown of Kir2.1 reversed the effect of MC4R deficient on energy balance and insulin resistance

**Fig. 6:**
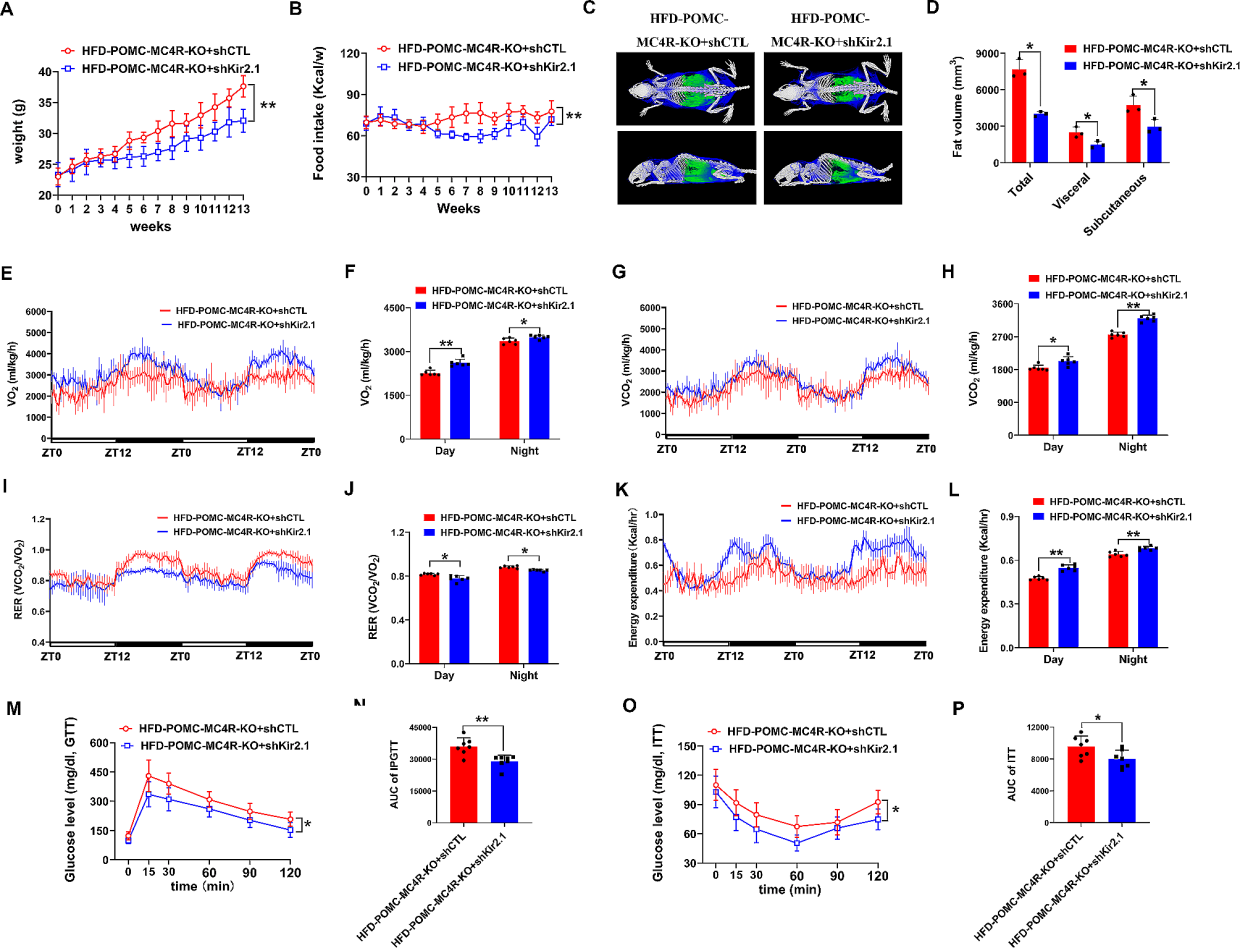
Knockdown of Kir2.1 reversed the effect of MC4R deficiency on energy balance and insulin resistance in HFD-treated mice. (**A**) Body-weight in MC4R-ko mice injected with AAV-Kir2.1 or sh-NC. (**B**) Food intake in MC4R-ko mice injected with AAV-Kir2.1 or sh-NC. (**C** and **D**) Fat mass at 21 wk old. (**E** and **F**) O_2_ consumption (VO_2_), (**G** and **H**) CO_2_ production, and (**I** and **J**) RER of the mice fed an HFD. (**K** and **L**) Energy expenditure was evaluated of the mice. (**M** and **N**) GTT (**M**) and the AUC of GTT (**N**) of 21-wk-old mice. (**O** and **P**) ITT (**O**) and the AUC of ITT (**P**) of 21-wk-old mice. *P < 0.05, **P < 0.01. Two-tailed Student’s *t* test was used for comparison for two groups

**Fig. 7:**
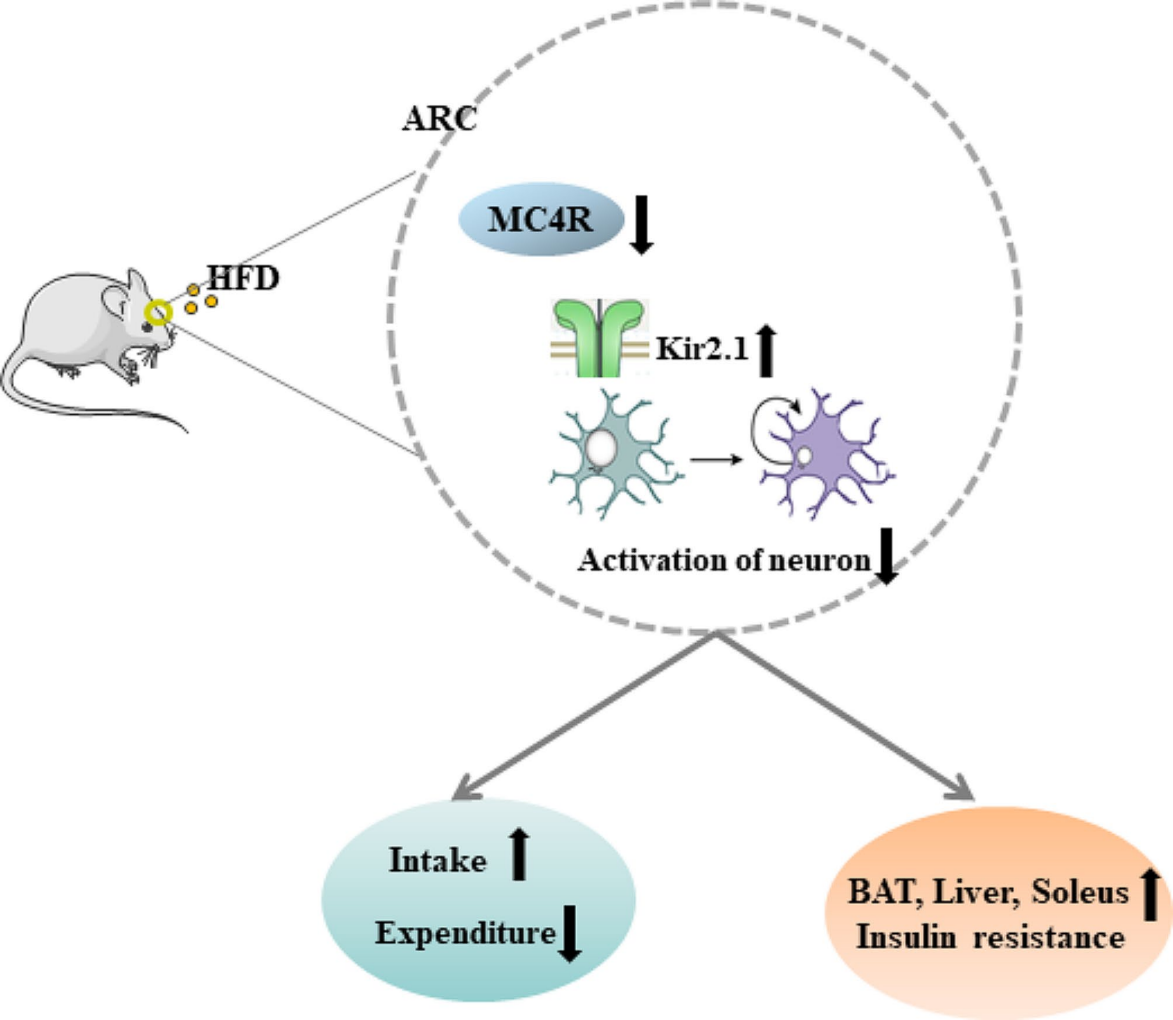
Graphical illustration of hypothalamic POMC neuron-specific knockout of MC4R affects insulin sensitivity by regulating Kir2.1

To assess the response of Kir2.1 in POMC-MC4R^flox/flox^ mice, we then used mice with sh-Kir2.1 vector injections to ARC of POMC-Cre mice to assess the effect of Kir2.1 on energy balance and insulin resistance in mice. Significantly lower body weight and food intake was observed in POMC-MC4R^flox/flox^ mice injected with sh-Kir2.1 compared to POMC-MC4R^flox/flox^ mice injected with sh-NC (Fig. 6A-B). The fat mass was significantly reduced following kir2.1 knockdown (Fig. 6C-D). Moreover, deletion of kir2.1 led to elevated oxygen consumption (Fig. 6E-F) and CO_2_ release (Fig. 6G-H), while reduced RER (Fig. 6I-J). The energy expenditure was highly increased after Kir2.1 knockdown (Fig. 6K-L). Additionally, Kir2.1 knockdown mice are less insulin resistance than the control mice, as indicated by decreased GTT level and AUC of the GTT (Fig. 6M-N). Consistent with these changes, knockdown of Kir2.1 also significantly reduced ITT level and AUC of the ITT (Fig. 6O-P). Together these data suggest a role for Kir2.1 in MC4R-expressing neurons in potentiating energy balance within the ARC at baseline (Fig. 7).

## Discussion

Since the discovery of the melanocortin system and its satiety promoting action via MC4R, POMC neurons have been considered as key drivers of cessation of feeding (Quarta et al. 2021). However, our findings unmasked an overlooked, albeit previously proposed role for hypothalamic POMC neurons in energy homeostasis and insulin resistance. Specifically, we found that POMC neuronal activation is indispensable for energy homeostasis and insulin resistance triggered by MC4R knockdown-induced Kir2.1 activation in the state of HFD. We have identified central neural as a mechanism by which MC4R-deficient mice remain protected from energy imbalance and insulin resistance. Knockdown of central neural Kir2.1 from MC4R-deficient mice restored the insulin resistance of MC4R-deficient mice.

Hypothalamus-controlled energy balance is complex and likely relies on diverse spatial sites (Kühnen et al. 2019). A recent study found that regulation of the firing activity of MC4R-expressing neurons in the PVN of the hypothalamus could be mediated by ligand-induced coupling of the MC4R to close (by means of α-MSH) or open (by means of AgRP) the inward rectifying potassium channel (Anderson et al. 2019). Restoring expression of MC4Rs specifically in the lateral hypothalamic nucleus improves glucose intolerance in obese MC4R-null mice through bilateral interscapular brown adipose tissue denervation without affecting body weight or circulating insulin levels (Morgan et al. 2015). POMC is the common precursor of many neuropeptides, including α-MSH, beta-endorphin and adrenocorticotropic hormone, which contribute to energy metabolism (Zhan 2018). Furthermore, POMC neurons can receive peripheral signals, such as leptin, insulin, adiponectin and glucagon-like peptide-1, thus modulating glucose metabolism through its corresponding receptors (Quarta et al. 2020). Recent study demonstrated that POMC neurons expressing the leptin receptor and POMC neurons expressing glucagon like peptide 1 receptor exhibit a specific anatomical distribution within the ARC and differentially express receptors for energy-state communicating hormones and neurotransmitters, resulting in differential ability to suppress feeding (Biglari et al. 2021). Thus, only a holistic approach that integrates the intrinsic cellular properties of POMC neurons with their spatial position (or microarchitecture) in the brain and their sensitivity to afferent signals can uncover how the activity of specific POMC neuronal subsets translates into specific behavioral or metabolic effects. Neuronal retrograde tracing identifies the ARC area as the primary target of MC4R-expressing neuron (Bagnol et al. 1999). Overexpression of MC4R in the ARC region may link to metabolic disorders of induced polycystic ovary syndrome and energy balance stimulation in the rats (Nooranizadeh et al. 2018; Sarvestani et al. 2015). Besides that, MC4R expressing neurons of the ARC are the POMC neurons, the activation of which reduces food intake behaviors (Tooke et al. 2019). Therefore, we focused on a specific action of ARC region. MC4R deficiency was induced since birth mice to account for the confounding effects of developmental or compensatory changes on glucose homeostasis. Whether chowing or fasting, MC4R has effects on insulin sensitivity and insulin resistance. But fasting has a bigger impact. Considering that the HFD and the normal chow used in the present study have different mix of micronutrients, and that micronutrients regulated insulin resistance in mice (Yang et al. 2023), the micronutrients in HFD may have synergistically contributed to increased insulin resistance in mice. Furthermore, MC4R deletion resulted in an increase in the RER, but decreased EE. This could be for the following two reasons: 1) The significantly reduced physical activity of MC4R deletion mice could be the direct cause of the decline in EE. Changes in metabolism led the body to store more energy as fat instead of releasing it as heat, resulting in fat accumulation. 2)MC4R deletion mice may be more efficient at carbohydrate metabolism, suggesting that while maintaining the energy supply required for living activities, they produce less total energy expenditure overall and have a higher RER.

The neural connection between the hypothalamus and the BAT, liver, and soleus plays an important role in the CNS control of systematic glucose homeostasis and insulin resistance (Henningsen and Scheele 2021; Pozo and Claret 2018; Uyama et al. 2004). Here, we found that knockout of MC4R downregulated levels of p-IR and p-AKT, two key components in the insulin signaling pathways (Boucher et al. 2014; Sharma and Dey 2021), in BAT, liver and soleus, demonstrating decreased insulin sensitivity among those three organs. Therefore, we speculated that central nervous system plays a vital role in MC4R regulates insulin sensitivity of peripheral organs.

With 13-week HFD feeding, the number of c-Fos-expressing neurons has little change. However, MC4R deficiency largely eliminated c-Fos expression in ARC POMC neurons, suggesting that MC4R deficient greatly reduced the response of ARC POMC responsiveness. The expression of Kir2.1 greatly blunted the responsiveness, suggesting that the action of Kir2.1, which likely locks the neuron activity at respective elevated or reduced levels, may override the action induced by stressors and hormones.

Transcriptome analysis found that the expression of Kir2.1 was down-regulated in MC4R overexpression mouse hypothalamic GT1-7 cells. It is reported that one member of inwardly rectifying potassium channels family, ATP-regulated potassium (KATP) channel complexes of inward rectifier potassium channel (Kir) 6.2 regulate glucose homeostasis by regulating pancreatic islet β-cell membrane potential, calcium influx, and insulin secretion (Raphemot et al. 2014). Kir2.1 functions as intracellular trafficking to cross-talk with the insulin-like growth factor receptor signaling pathway, insulin receptor, as well as lysosomal degradation (Park et al. 2020). Hyperactive Kir2.1 channels in human β-cells may lead to reduced insulin secretion (Riz et al. 2015). These findings suggested a potential role of Kir2.1 in insulin resistance. Here, we observed a 50–65% increment in Kir2.1 levels in MC4R-deficient mice. Although this increment in Kir2.1 level is sufficient to cause reduced appetite and insulin resistance in MC4R-deficient mice, we speculate that hypothalamus-specific Kir2.1 knockout mice will exhibit significantly lower energy than that observed in MC4R-deficient mice. Regulation of G protein-coupled inwardly rectifying potassium (GIRK) channels by GPCRs via the G protein βγ subunits has been well characterized (Carrington et al. 2018). However, Litt et al. (Litt et al. 2018) reported that MC4R regulates Kir7.1 activity through a mechanism independent of Gs. Besides that, GPCR-mediated reduction of Kir7.1 glycosylation in HEK293T cells decreased channel activity (Carrington et al. 2018). Whether MC4R regulates Kir2.1 dependent or independent of Gs, as well as the mechanisms underlying MC4R regulation of Kir2.1 activity is unknown. Future studies are necessary to answer these questions.Given that some Cre strains are also active in other tissues, and that POMC precursors give rise to AgRP/NPY neurons and also to other neurons in the hippocampus and amygdala, in situ hybridization to look at mRNA levels of MC4R can better explain the effect of POMC neuron-specific knockout of MC4R on insulin sensitivity.

In summary, this study demonstrates that MC4R selectively in the ARC contributes to energy balance and insulin resistance by reducing the activation of POMC neuron via hypothalamus Kir2.1. These results demonstrate that hypothalamic POMC neuron-specific knockout of MC4R affects energy balance and insulin sensitivity by regulating Kir2.1, thus providing a novel insight on the mechanism underlying HFD. This novel mechanism has important implications to prevalent investigations focusing on the role of neuron activity levels in the regulation of feeding and obesity development.

### Electronic supplementary material

Below is the link to the electronic supplementary material.

Supplementary Material 1

Supplementary Material 2

Supplementary Material 3

Supplementary Material 4

Supplementary Material 5

## Data Availability

The datasets used and/or analyzed during the current study are available from the corresponding author on reasonable request.

## Abbreviations

**AgRP**: Agonist agouti-related protein
**α-MSH**: Agonistα-melanocyte-stimulating hormone
**ARC**: Arcuate nucleus
**Co-IP**: Co-immunoprecipitation assays
**GTT**: Glucose tolerance test
**HFD**: High fat diet
**IR**: Insulin resistance
**ITT**: Insulin tolerance test
**MC4R**: Melanocortin-4 receptor
**POMC**: Proopiomelanocortin
**RER**: Respiratory exchange ratio
