# Supplementary figures and images for: Hypothalamic POMC neuron-specific knockout of MC4R affects insulin sensitivity by regulating Kir2.1
Source: Mol Med. 2024 Mar 6;30:34. doi: 10.1186/s10020-024-00804-z (PMC10918880; doi:10.1186/s10020-024-00804-z)

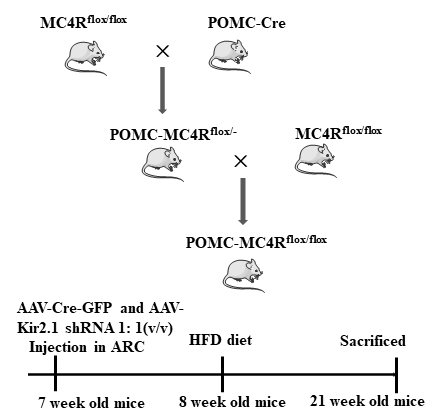

Supplement: Supplementary file 1 — Supplementary Material 1 [file 10020_2024_804_MOESM1_ESM.tif]

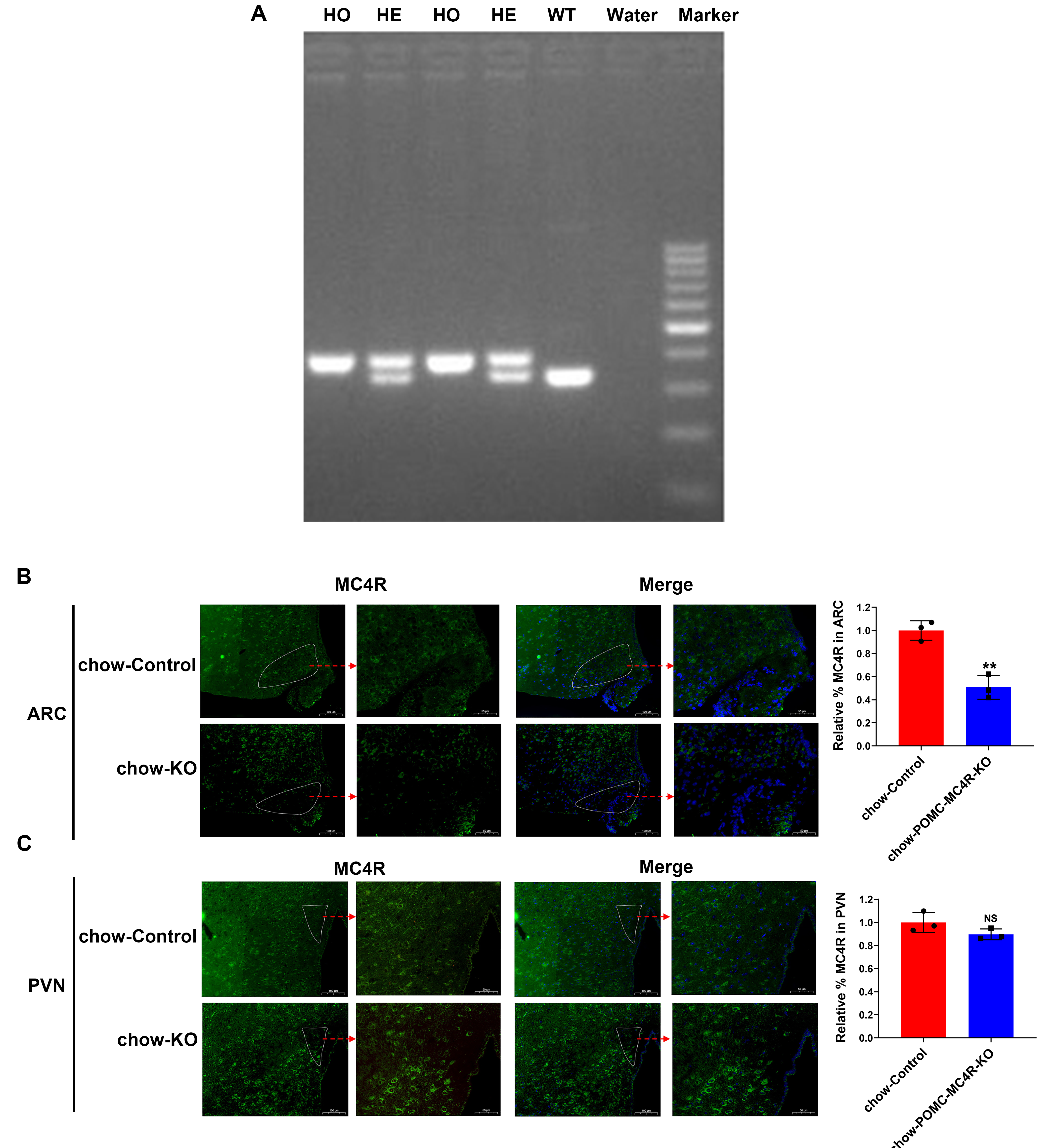

Supplement: Supplementary file 2 — Supplementary Material 2 [file 10020_2024_804_MOESM2_ESM.tif]

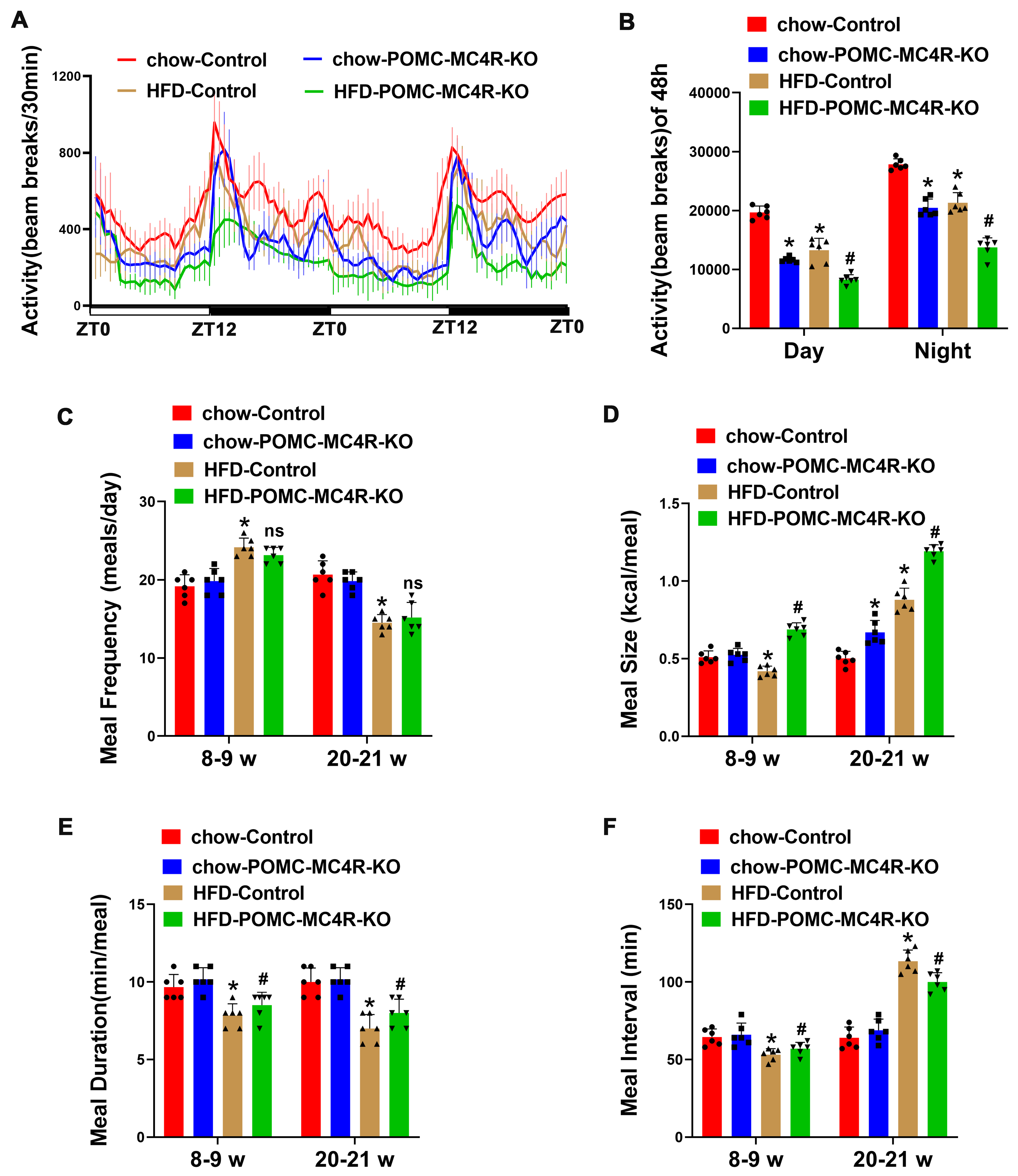

Supplement: Supplementary file 3 — Supplementary Material 3 [file 10020_2024_804_MOESM3_ESM.tif]

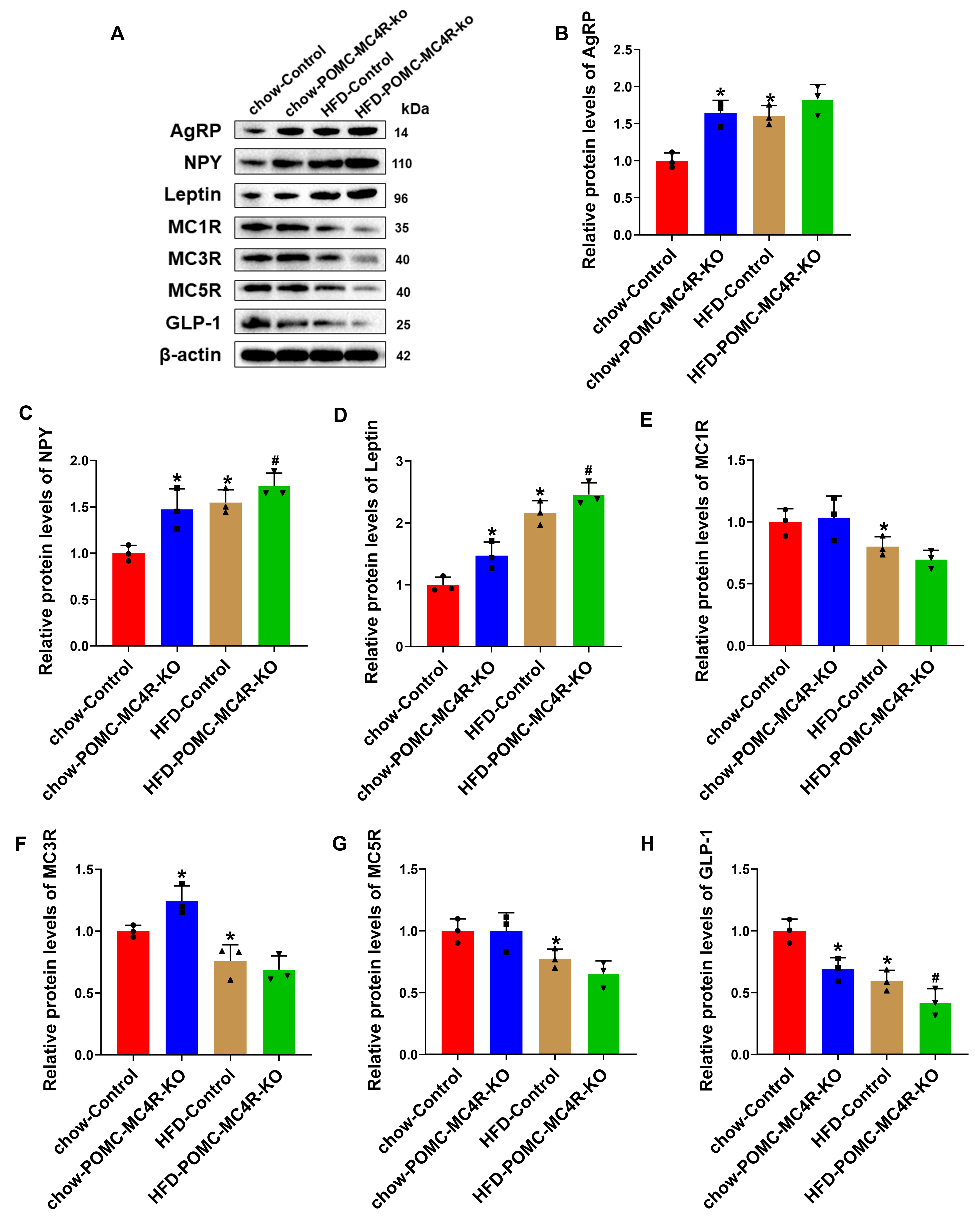

Supplement: Supplementary file 4 — Supplementary Material 4 [file 10020_2024_804_MOESM4_ESM.tif]

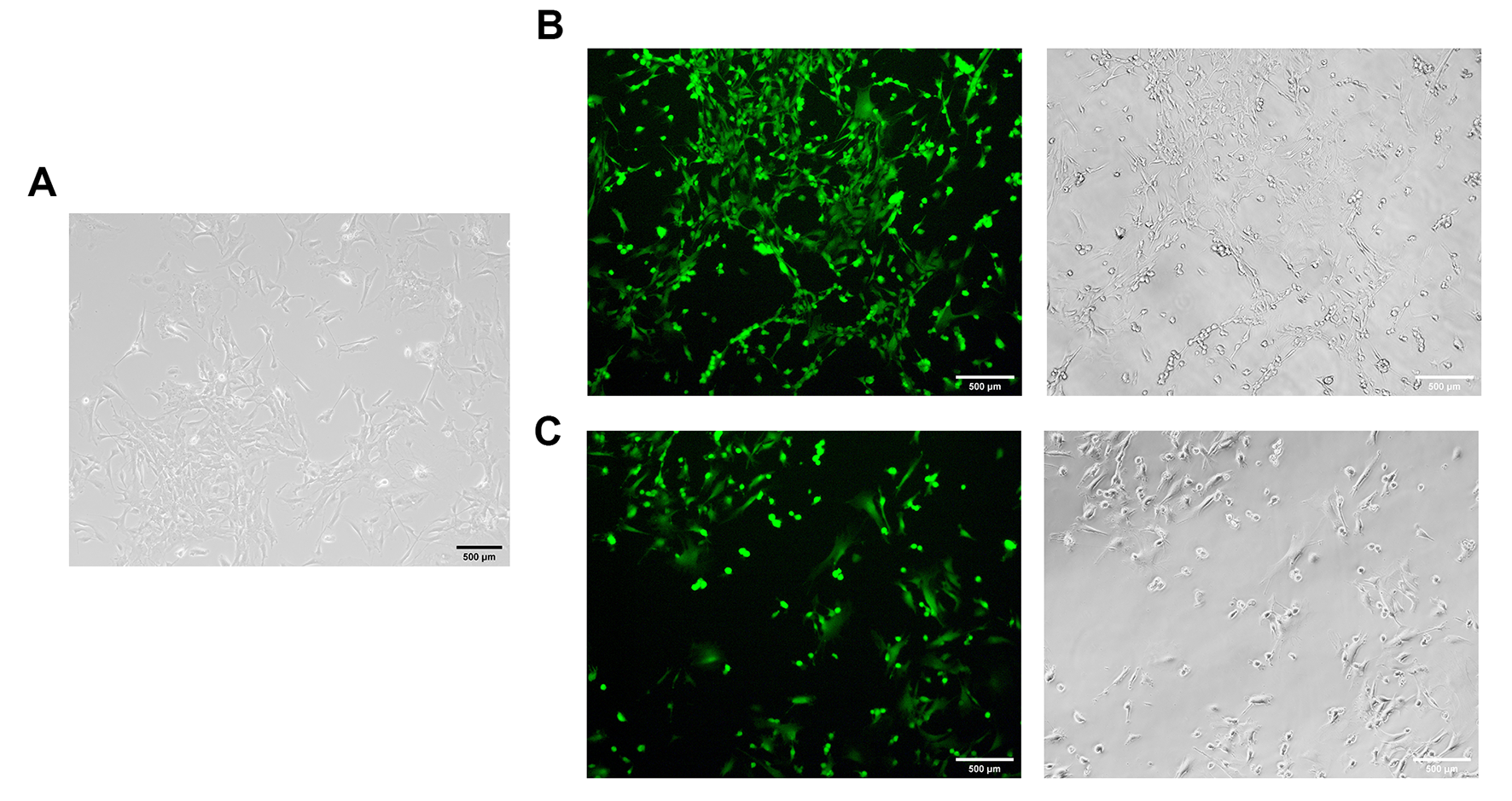

Supplement: Supplementary file 5 — Supplementary Material 5 [file 10020_2024_804_MOESM5_ESM.tif]
